# Development of an SNP Assay for Marker-Assisted Selection of Soil-Borne *Rhizoctonia solani* AG-2-2-IIIB Resistance in Sugar Beet

**DOI:** 10.3390/biology11010049

**Published:** 2021-12-29

**Authors:** Samathmika Ravi, Mahdi Hassani, Bahram Heidari, Saptarathi Deb, Elena Orsini, Jinquan Li, Christopher M. Richards, Lee W. Panella, Subhashini Srinivasan, Giovanni Campagna, Giuseppe Concheri, Andrea Squartini, Piergiorgio Stevanato

**Affiliations:** 1 Department of Agronomy, Animals, Natural Resources and Environment-DAFNAE, University of Padova, 35020 Legnaro, PD, Italy; samathmikaravi@gmail.com (S.R.); saptarathideb@gmail.com (S.D.); giuseppe.concheri@unipd.it (G.C.); squart@unipd.it (A.S.); 2 Sugar Beet Seed Research Department, Hamedan Agriculture and Natural Resources Research and Education Centre, AREEO, Hamedan 65519, Iran; m.hasani@areeo.ac.ir; 3 Department of Plant Production and Genetics, School of Agriculture, Shiraz University, Shiraz 7144165186, Iran; bheidari@shirazu.ac.ir; 4 Strube Research GmbH & Co. KG, 42651 Söllingen, Germany; e.orsini@strube.net (E.O.); j.li@strube.net (J.L.); 5 USDA-ARS, National Laboratory for Genetic Resources Preservation, Fort Collins, CO 80521, USA; chris.richards@usda.gov (C.M.R.); lee.panella@Colostate.edu (L.W.P.); 6 Institute of Bioinformatics and Applied Biotechnology, Bengaluru 560100, India; ssubha@ibab.ac.in; 7 COPROB, 40061 Minerbio, BO, Italy; giovanni.campagna@coprob.com

**Keywords:** *Rhizoctonia solani*, sugar beet, plant breeding, marker-assisted selection, RAD sequencing, SNP discovery, PCA biplot, SNP validation

## Abstract

**Simple Summary:**

Sustainable breeding of sugar beet against *Rhizoctonia solani* relies on the continuous identification of resistance genes to allow their integration into new and modern cultivars. Better control of the disease may thus be achieved by a combination of tolerant or resistant cultivars selected based on molecular markers such as SNPs. The utility of one such marker, RsBv1 (Chromosome 6, 9,000,093 bp, C/T), located in an ADP-ribosylation factor and associated with *Rhizoctonia* resistance resulting from validation of three geographically diverse plant materials is reported.

**Abstract:**

*Rhizoctonia solani*, causing *Rhizoctonia* crown and root rot, is a major risk to sugar beet (*Beta vulgaris* L.) cultivation. The development of resistant varieties accelerated by marker-assisted selection is a priority of breeding programs. We report the identification of a single-nucleotide polymorphism (SNP) marker linked to *Rhizoctonia* resistance using restriction site-associated DNA (RAD) sequencing of two geographically discrete sets of plant materials with different degrees of resistance/susceptibility to enable a wider selection of superior genotypes. The variant calling pipeline utilized SAMtools for variant calling and the resulting raw SNPs from RAD sequencing (15,988 and 22,439 SNPs) were able to explain 13.40% and 25.45% of the phenotypic variation in the two sets of material from different sources of origin, respectively. An association analysis was carried out independently on both the datasets and mutually occurring significant SNPs were filtered depending on their contribution to the phenotype using principal component analysis (PCA) biplots. To provide a ready-to-use marker for the breeding community, a systematic molecular validation of significant SNPs distributed across the genome was undertaken to combine high-resolution melting, Sanger sequencing, and rhAmp SNP genotyping. We report that RsBv1 located on Chromosome 6 (9,000,093 bp) is significantly associated with *Rhizoctonia* resistance (*p* < 0.01) and able to explain 10% of the phenotypic disease variance. The related SNP assay is thus ready for marker-assisted selection in sugar beet breeding for *Rhizoctonia* resistance.

## 1. Introduction

Sugar beet cultivation, contributing to ~20% of the world’s sugar production, is distressed by different pathogens. One such fungal pathogen is the soil-borne fungus, *Rhizoctonia solani* which is recurrent and perennial in all sugar beet production areas and causes the typical root and crown rot. Symptoms can range from localized brown to black lesions on the root surface to complete rotting of the root. Symptoms are also characterized by sudden wilting of the leaves and black lesions on the petioles attaching to the crown area. The extent of the damage it causes and the yield losses vary in sugar beet farms and are considerable and dramatic [1,2,3,4]. Specifically, the loss in terms of recoverable white sugar is estimated at 50–60% and sometimes results in complete crop failure too [1,2,3].

One of the most direct and economical management strategies is breeding varieties for genetic resistance. Growers are strongly advised to control the disease by combining agronomic measures such as long crop rotations (alternating crops year-to-year) and using resistant varieties adapted to the production areas [3,5]. In the late 1960s, an intensive breeding program to develop resistance to *R. solani* was initiated and substantial improvements in *Rhizoctonia* resistance were achieved with germplasm releases FC701 and FC702 [6,7,8]. In 1999, two additional sugar beet accessions, FC709-2 (Reg. no. GP-200, PI 599668) and FC727 (Reg. no. GP-201, PI 599669), were introduced by the USDA-ARS, Fort Collins, CO, in cooperation with the Beet Sugar Development Foundation, Denver, CO [9]. These two non-O-type, multigerm, and pseudo-self-fertile lines provided a high level of resistance to root-rotting strains (AG-2-2) of *R. solani*. Yet another accession, FC 712 (Reg. no. GP-97) was registered in 1985 as a source of resistance to *R. solani* [10]. Along with the identification of different sources of resistances, studies have shown that male-sterile cytoplasm has no influence on the resistance and that the development of triploid hybrids should be advantageous in breeding programs of *Rhizoctonia* resistance [11]. Consequently, relentless efforts to improve the resistance have led to the development of many *Rhizoctonia* resistant lines in sugar beet [12,13,14,15,16,17,18]. However, the resistance remains incomplete and is often accompanied by decreased yield potential [19,20]. Newly, a significant negative association between yield and resistance (R) genes was observed in new and modern soybean cultivars in comparison to the wild and landraces of soybean. Here, it was hypothesized that the association between lower R gene content and yield could be due to the preparedness of plants for defense through activation of immune pathways compromising the plant growth through hormone signaling pathways resulting in subsequent vigor and yield losses [21]. Although partial dominant genes [7] and many QTLs [22] have been identified for disease resistance, this trait is quantitative, and the contribution of minor QTLs and small genomic regions cannot be ruled out. Very recently, the overexpression of major latex proteins (MLPs) in resistant genotypes of sugar beet in response to *Rhizoctonia solani* was shown [23]. This class of defense genes could have the potential for molecular resistance breeding.

The development of disease resistance-linked markers can substantially aid the breeding programs for *Rhizoctonia* resistance. Single nucleotide polymorphisms (SNPs) identified through DNA sequencing are the most robust and abundant co-dominant markers for mapping genes affecting traits of interest in crop plants [24,25]. SNP markers and related SNP allelic discrimination assays are well suited for screening many individuals in segregating and natural populations being compatible with high throughput detection technologies. Despite its popularity in breeding for a range of valuable traits, no effort has been devoted to the identification of SNP markers associated with *Rhizoctonia* resistance in sugar beet germplasm. The increasing use of affordable and high-throughput technologies such as next-generation sequencing (NGS) allows the discovery of panels of SNPs appropriate for the identification of allelic combinations linked to agronomic traits that are functionally advantageous in breeding programs [26,27]. Particularly, reduced representation sequencing methods such as genotyping-by-sequencing (GBS) and restriction site-associated digestion sequencing (RAD-seq) have devised strategies to specifically target homologous DNA regions. Such conserved genomic regions that are important for cross-over and meiotic recombination result in the identification of true SNPs, providing a favorable advantage to progeny and covering a broader range of genetically diverse materials [28]. While these methods form the basis for the identification of suitable targets, functional correlation of candidate markers for further translation call for the availability of the reference genome becomes pertinent in studies involving marker development for crop improvement, and the genomic resources available for sugar beet are paramount [29,30].

In this study, we report the discovery and validation of useful SNP associated with *Rhizoctonia* resistance tested in several international sugar beet germplasm. Firstly, we carried out restriction site-associated DNA (RAD) sequencing on geographically diverse breeding materials, from Iran and Germany, with different degrees of resistance/susceptibility. Subsequently, a joint bioinformatics analysis of the sequencing data along with efficient bioinformatics pipelines resulted in the identification of contributing loci distributed across the sugar beet genome. Finally, a wide molecular validation strategy on diverse plant material was used for cross-validation of the identified SNPs in many individual sugar beet plants and worldwide reference *Rhizoctonia*-resistant cultivars. Our marker assay resulted in the development of RsBv1 for marker-assisted breeding programs of disease resistance and cultivar release.

## 2. Materials and Methods

### 2.1. Plant Material

#### 2.1.1. Germplasm for Rhizoctonia Phenotyping and SNP Discovery

The plant material provided by the Sugar Beet Seed Institute (SBSI), Iran, comprised 42 S1 (self-pollinated), 27 pollinator lines, 18 three-way cross hybrids, and 5 single-cross hybrids (Table 1). This germplasm was used for both phenotyping and RAD sequencing.

The second germplasm tested was plant material provided by Strube Research, Germany, and comprised a collection of 62 breeding lines with a broad genetic diversity in terms of *Rhizoctonia* resistance/susceptibility. Ten lines including the five highest resistance and five highest susceptible lines of this breeding material were sequenced using RAD sequencing.

#### 2.1.2. Germplasm for SNP Validation

The plant material provided by USDA consisted of a highly resistant check (FC709-2), a moderately resistant check (FC703), and a susceptible check (FC901/C817). The rating for symptoms (Ruppel score between 0 and 7) was determined based on field experiments of three replicated plots consisting of 8 plants each from the year 2018. The phenotyping data are also shared in Supplementary Materials Table S2. These plants were used for validation of the identified RsBv1 by Sanger sequencing and rhAmp genotyping. Additional plant material consisting of 54 resistant and 50 susceptible checks of *Rhizoctonia* were provided by the SBSI, Iran. These were used for rhAmp genotyping of RsBv1. Melindia (KWS, Germany), Moliere (Strube Research, Germany), and Octopus (SESVanderHave, Belgium) provided by COPROB, Italy were used as resistant checks. A CMS line and an O-type from susceptible background provided by the Department of Agronomy, Animals, Natural Resources and Environment (DAFNAE), Padova University, Italy, used for screening through HRM and Sanger sequencing and rhAmp validation of RsBv1. Three plants per line, for a total of 186 plants belonging to the 62 breeding lines of Strube Research, were randomly selected for rhAmp validation of RsBv1.

### 2.2. Phenotyping for Rhizoctonia Resistance

#### 2.2.1. Phenotyping of SBSI Germplasm, Iran

The experimental site was located at the Hamedan Agricultural and Natural Resources and Education Centre, Hamedan, Iran and the field trials were carried out in the year 2017–2018. Seeds of each genotype were directly sown in two 2-m long rows. The soil texture was sandy loam enriched. Fertilizer requirement was determined based on soil analysis. Weed control was performed manually and no herbicide was used. Plants in each plot were thinned down to 20 plants in each row at the four to the six-leaf stage of sugar beet growth. *Rhizoctonia* inocula were prepared from a highly aggressive isolate of *R. solani* (AG-2-2-IIIB (R9)). The isolate selected for this study was provided by SBSI. An inoculum of the fungus *R. solani* was developed by growing strain R9 on corn grains for 3 weeks at 25 °C. Seven weeks after sowing, inoculation of plants was performed through the addition of six infested corn grains to the soil around the plant crown area following a previously described method [31]. In the first week after inoculation, irrigation was performed every day. Thereafter, plants were well watered based on standard irrigation regime until the appearance of the disease symptoms. No fungicides were applied. Six weeks after the start of the inoculation, a rating for *Rhizoctonia* symptoms was performed for each plant. The commercial hybrids Jolgeh introduced by SBSI, Iran, and Kermit (Maribo Seed Factory, Denmark) were used as susceptible and resistant checks, respectively. Rating disease symptoms on the roots were performed following a nine-class disease scale [2]. Roots in each plant were lifted and gently washed and scored on a scale of 1 to 9, with 1 = no rot and 9 = entire plant dead. The scoring scale has been presented in Supplementary Materials Figure S1.

#### 2.2.2. Phenotyping at Strube Research, Germany

A collection of 62 lines chosen from the O-type and pollinator breeding programs of Strube Research were phenotyped in a greenhouse using 50 plants per line in summer 2019. *Rhizoctonia* inocula were prepared from a highly aggressive isolate of *R. solani* (AG-2-2, IIIB) developed on barley grains. Eight weeks after sowing, inoculation of plants was performed through the addition of infested barley grains to the soil around the plant crown area following an internal protocol. The experiment was set up as an incomplete randomized block design with two susceptible and two resistant checks included. A total of 3370 plants were tested in two greenhouses on seven tables and 168 trays each consisting of 20 plants where at least one check was included in the tray. The experiment was conducted in summer under controlled conditions with the following parameters: day temperature: 24 °C, night temperature: 20 °C, air humidity: 80%, air humidity during inoculation: 100%, photoperiod 6:00 to 22:00, light intensity: 15,000 lux). No fungicides were applied. Rating disease symptoms occurred two weeks after inoculation on the roots of single plants based on the percentage of infected roots. The scoring scale was adopted from a recently published study [32]. Adjusted means for the *Rhizoctonia* scoring were calculated for each line with a mixed linear model to correct the effects from greenhouses, tables, and trays. The means for percentage infection were then converted to the ten-class disease scale [2].

### 2.3. DNA Isolation and RAD Sequencing

Leaves from plant materials listed in Table 1 were collected to extract DNA representing the material from SBSI. For the material from Strube Research, 70 plants from 5 extremely resistant lines (SR-R1, SR-R2, SR-R3, SR-R4, and SR-R5) and 110 plants from 5 extremely susceptible lines (SR-S1, SR-S2, SR-S3, SR-S4, and SR-S5) were selected for DNA extraction. Genomic DNA was obtained using a modified CTAB DNA extraction method [33]. The quality of the extracted DNA was checked on a 0.8% agarose gel and quantification of the same was done using a Qubit 4.0 fluorimeter (Thermo Fisher Scientific, Waltham, MA, USA). The DNA samples were subjected to a restriction site-associated DNA technology (RAD-seq) [34] with a HiSeq 2000 sequencing system using 150-bp single-end sequencing (Illumina Inc., San Diego, CA, USA).

### 2.4. Data Analysis for SNP Discovery

Variant calling was performed individually on the two datasets from SBSI and Strube Research and adapted from the earlier published method [35]. The pipeline uses fastqc (version 0.11.5) for quality check of reads, bowtie2 (version 2.3.5.1) for mapping reads to the reference, samtools (version 1.9), Picard-tools (version 2.18.12), samtools (version 1.9) mpileup, and bcftools (version 1.9) to extract variants. The resulting vcf files were filtered using bcftools (version 1.9). The EL10 sugar beet reference genome (NCBI PRJNA413079) was used as reference genome [30]. The base file for all the bioinformatics analyses described was the merged vcf file containing genotypes of the identified markers. For the genome-wide association analyses, the genotypes for each of the SNP markers were reduced to allele frequencies using the genetics package [36] in R statistical program. Statistical Fisher test of independence using custom R scripts was then conducted to test the difference of partial association of genotypes in two strata of contingency tables. Manhattan plots were developed using the CMplot library in R [37]. A subset of highly associated SNPs commonly occurring in both datasets (*p* < 0.05) was selected as preliminary candidates. The genotype matrix of 0 (homozygous reference allele), 1 (heterozygous allele), 2 (alternate homozygous allele) was analyzed to assess the contributions of the selected SNPs in the separation of phenotypes using PCA biplots. They were developed using the FactoMineR package in R [38] and further used for downstream molecular validation.

### 2.5. Molecular Validation

#### 2.5.1. High-Resolution Melting (HRM) Analysis

Flanking sequences of 150 bp around the target SNP were extracted using the bedtools getfasta tool (version 2.28.0). The list of sequences was given as input to the stand-alone version of the Primer3 software (version 4.0) to design primers with suitable amplicon lengths. The HRM analyses were carried out in 384 well plates on the QuantStudio 12K Flex (Life Technologies, Carlsbad, CA, USA). Reactions were carried out according to a previously described method [35]. The melt curve profiles were analyzed using the HRM software. 

#### 2.5.2. Sanger Sequencing

Flanking sequences of 250 bp around the target SNP were extracted using the bedtools getfasta tool (version 2.28.0). The list of sequences was given as input to the stand-alone version of the Primer3 software (version 4.0) to design primers with suitable amplicon lengths. The reaction setup and PCR cycling conditions were adopted from an earlier study from our lab [35]. The amplicons were run on a 1.5% agarose gel to verify the amplification. PCR products were purified using NucleoSpin Gel and PCR Clean-up (Macherey–Nagel, Bethlehem, PA, USA) and were sent for sequencing. The resulting sequences were analyzed using SnapGene 5.1.7 (Chicago, IL, USA) to generate multiple sequence alignments and chromatograms. 

#### 2.5.3. Genotyping

Validated SNPs from Sanger sequencing were used to design rhAmp assays (Integrated DNA Technologies, Coralville, IA, USA). Sequences of the SNP are presented in Supplementary Materials Table S3. Three hundred and forty-nine biological samples comprising material from Iran, Germany and USA were subjected to rhAmp genotyping, which was performed in 5 μL using 384-well plates, and low Rox was used as a passive reference dye. 5 ng of DNA was mixed with 2.65 μL of rhAmp Genotyping Master Mix, 0.25 μL of rhAmp SNP assay mix, and 1 μL of nuclease-free water. The thermal cycle parameters were adopted from Broccanello et al. (2018). 

## 3. Results

### 3.1. Phenotyping

**Figure 1:**
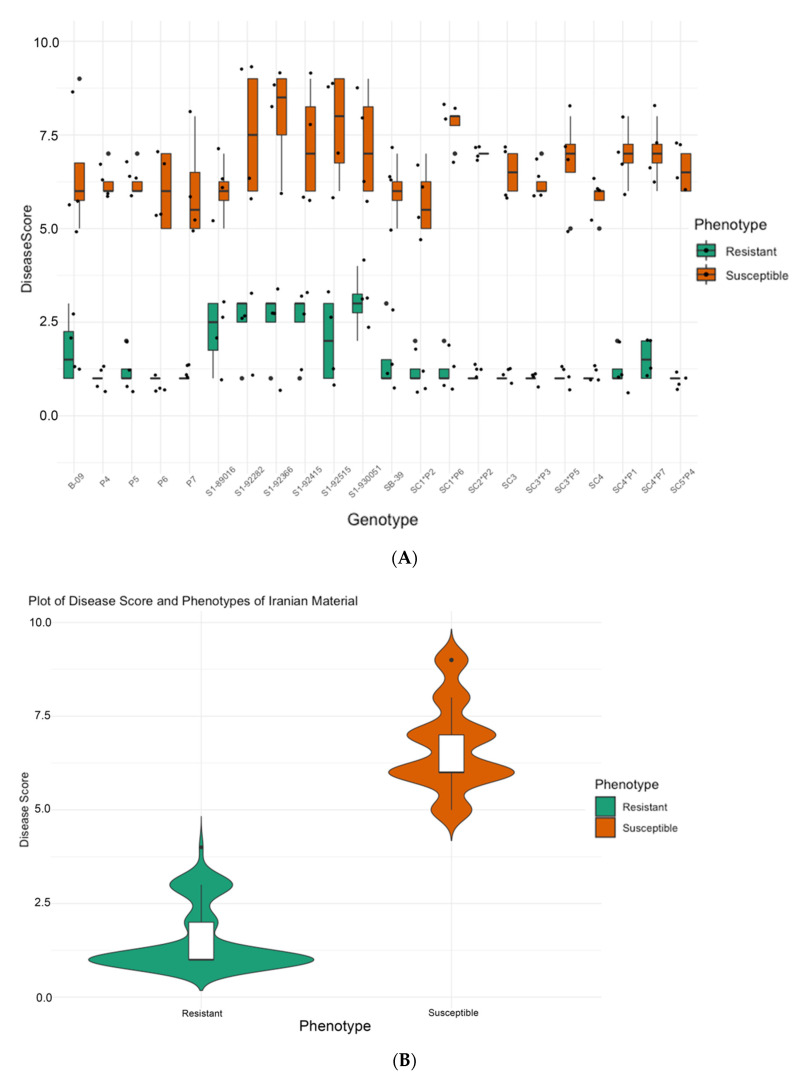
(**A**) Distribution of disease severity [2] scores across tested germplasm from SBSI which shows the range of variation for plants from each variety classified as resistant and susceptible. (**B**) Resistance trait summary plot of material grouped based on phenotype, the width of the shaded area can be correlated with the density of the data points.

Figure 1A summarizes observations of disease severity on the germplasm from SBSI comprising 8 self-pollinated genotypes (B-09, S1-89016, S1-92282, S1-92366, S1-92415, S1-92515, S1-930051, and SB-39), 8 hybrid genotypes (SC1*P2, SC1*P6, SC2*P2, SC3*P3, SC3*P5, SC4*P1, SC4*P7, SC5*P4), 4 pollinators (P4, P5, P6, P7), and 2 single-cross male sterile (SC3 and SC4). The resistant material had an average disease score of 1.52 ± 0.84 and the susceptible material showed an average rating of 6.68 ± 1.15 (Figure 1B).

**Figure 2:**
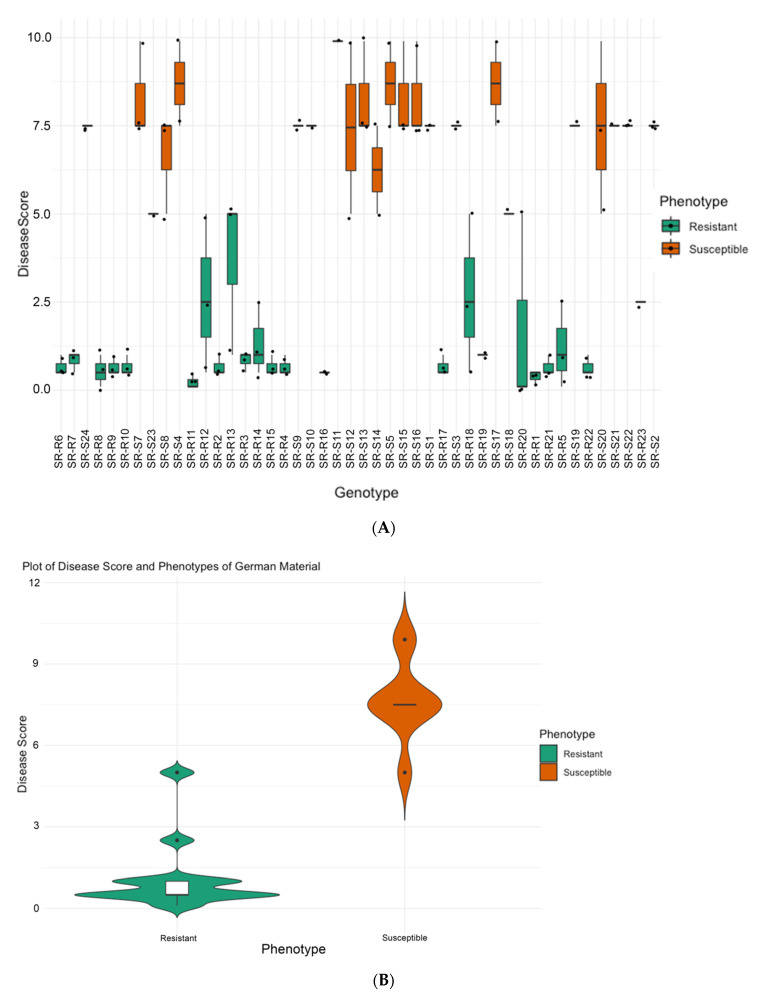
(**A**) Distribution of disease severity [2] scores across tested germplasm from Germany. The range of variation for plants from each variety classified as resistant and susceptible is shown. (**B**) Resistance trait summary plot of material from grouped based on phenotype. The width of the shaded area can be correlated with the density of the data points.

For the germplasm from Strube, Germany, the distribution of disease scores of two-three randomly chosen plants from 46 parental lines are shown in Figure 2A. The resistant group shows an average of 1.09 ± 1.06 disease severity whereas the susceptible group presented an average of 7.68 ± 1.40. The rating allowed us to separate the material as resistant and susceptible to proceed with the most resistant and most susceptible for sequencing detailed in Table 1 (SBSI) and described in the Materials and Methods for Germany.

### 3.2. SNP Discovery for Rhizoctonia Resistance

**Figure 3:**
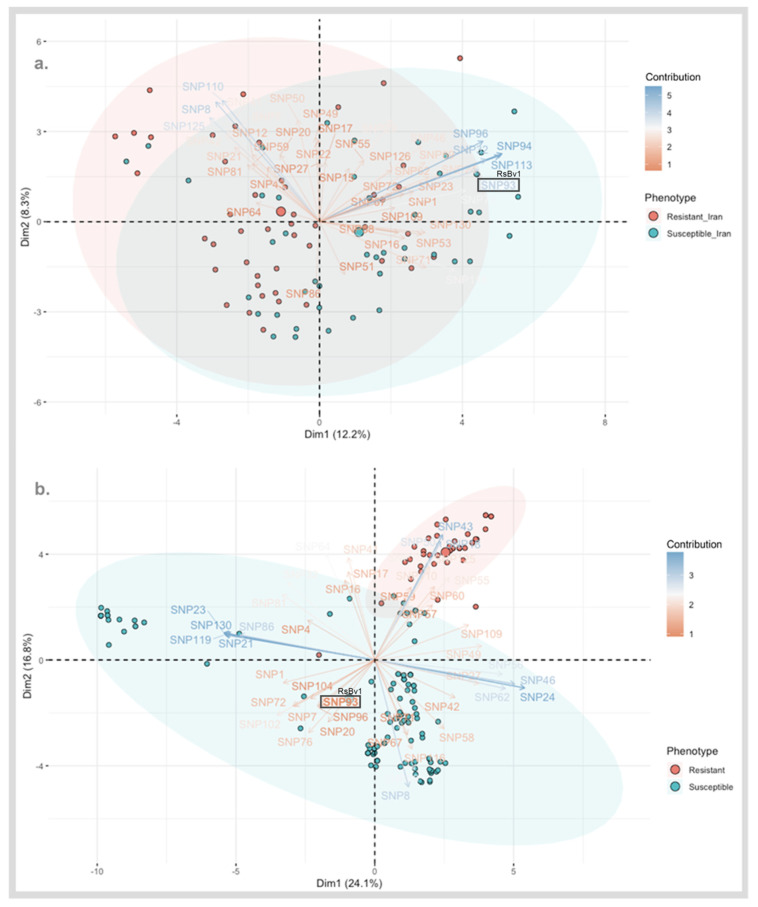
(**a**) PCA-biplot of selected 297 SNPs from the association analysis estimated 20.5% of phenotypic variation in SBSI germplasm. (**b**) PCA-biplot of selected 297 SNPs from the association analysis estimated 40.9% of phenotypic variation in Strube Research germplasm. Clusters of individuals on the same side of a given variable (SNP) have a higher value for the same (encoded as 0 for reference allele, 1 for heterozygous calls and 2 for alternate allele). For example, the contribution of RsBv1 in the opposite direction of resistant clusters is seen indicating that the majority of the resistant plants have the alternate allele.

The total number of raw SNPs upon mapping to EL10 reference genome were 15,988 and 24,439 for the SBSI and Strube Research germplasms, respectively. The association analysis resulted in the selection of candidate SNPs differing significantly in their allelic status and discriminating the sample groups (*p* < 0.05). A total of 297 significant SNPs mutually occurring between the two datasets (Iran and Germany) are shown using circular Manhattan in Supplementary Materials Figures S2 and S3. The PCA-biplot shows the contributions of the SNPs and the phenotypes (Figure 3). An important consideration here is the coding of SNPs (described in the methods section) and the number of interactions (SNPs * individuals). It is recommended as a practice to express the data as 0 and 2 for the homozygotes and 1 for the heterozygote [39]. For better visualization, the input data matrix can be altered by managing the number of SNPs and individuals [39]. In our case, the SNP numbers were filtered as described in the previous section.

In principle, similar interactions were anticipated between two points of the same kind (two samples or two SNPs). For two different vectors (a sample and a SNP), samples in each direction from the origin have positive interactions for SNPs in the same direction. Samples present in the opposite directions of SNPs have negative interactions for those SNPs. Thus, the influences are larger when a sample or SNP is farthest from the origin. Similarly, samples near the origin have the smallest influences from all SNPs and likewise for SNPs present near the origin.

The contributions of the individuals (sequenced samples of SBSI and Strube Research) and variables (significant SNPs) are shown in Figure 3. At a first glance, the separation of resistant and susceptible clusters can be seen which is a good validation of the phenotyping method. Figure 3A shows that the selected SNPs were able to account for 20.5% of the phenotypic variation. For example, a strong positive influence of SNP96, SNP94, SNP113, RsBv1, and SNP32 is seen on the susceptible material which is opposite to the resistant material. Similarly, a strong positive contribution of SNP110, SNP125, and SNP8 is seen on the resistant material, contrasting with the susceptible samples.

Figure 3B shows that the same set of filtered SNPs from the association analyses contributed to 40.9% of the phenotypic variation in the Strube germplasm. A good separation between resistant and susceptible phenotypes can be seen as shown by the red and blue ellipses. A strong influence of SNP43, SNP18, and SNP50 on the resistant cluster is seen. The confirmation of positive contribution of SNP96 and RsBv1 intermediate between the susceptible clusters can be seen (shown also in Figure 3A). In this way, SNPs explaining the same kind of behavior between phenotypes, such as RsBv1, can be considered as interesting targets related to resistance and for downstream validation. The sequences of SNP targets from the top 62 commonly occurring SNP targets are shared in Supplementary Materials Table S1.

### 3.3. Validation of SNPs Using High-Resolution Melting (HRM) Analysis and Sanger Sequencing

**Figure 4:**
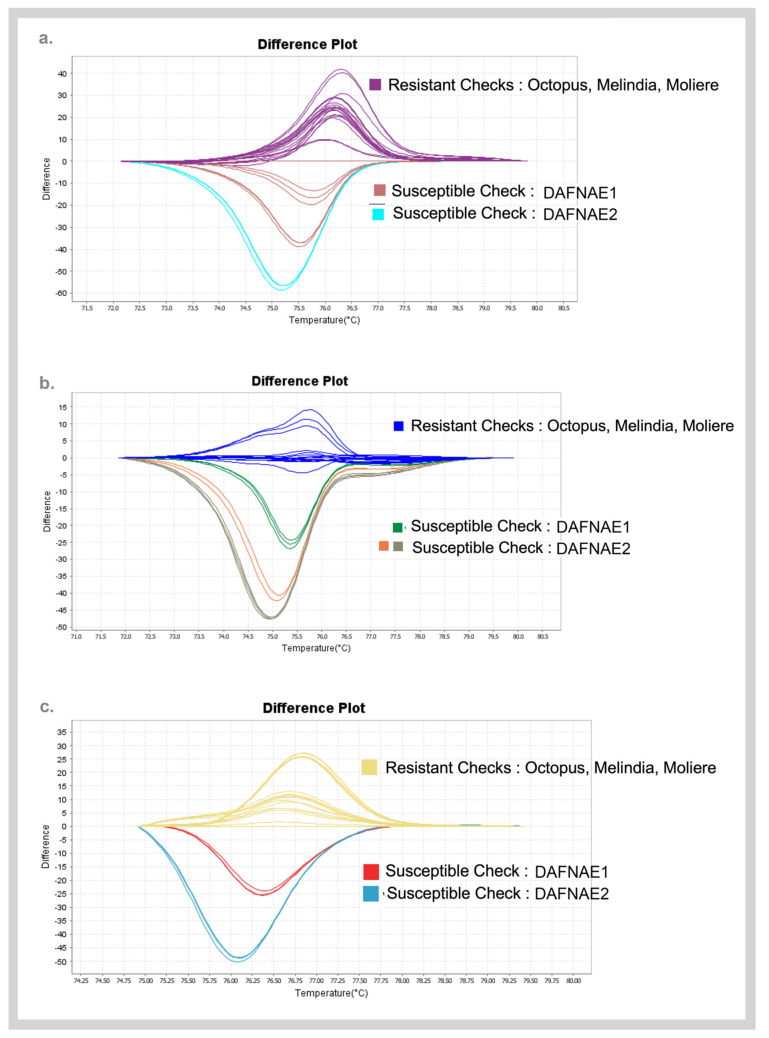
Melt curve profiles of best targets (**a**) SNP94 (Chr6), (**b**) SNP23 (Chr9), and (**c**) RsBv1 (Chr6) from high-resolution melting analyses discriminating the resistant and susceptible checks.

A subset of SNP candidates was studied for its contribution in discriminating resistance/susceptible phenotypes from the bioinformatics analysis that were subsequently screened using high-resolution melting (HRM) analysis. Figure 4 shows the representative melt curve profiles of SNP target sequences with the highest difference in the melting temperature between the contrasting phenotypes. The differences in the melt curves are due to differences in the sequence composition between resistant and susceptible checks which confirm the presence of the SNP. Convincing SNP targets from this screening were taken further for Sanger sequencing.

**Figure 5:**
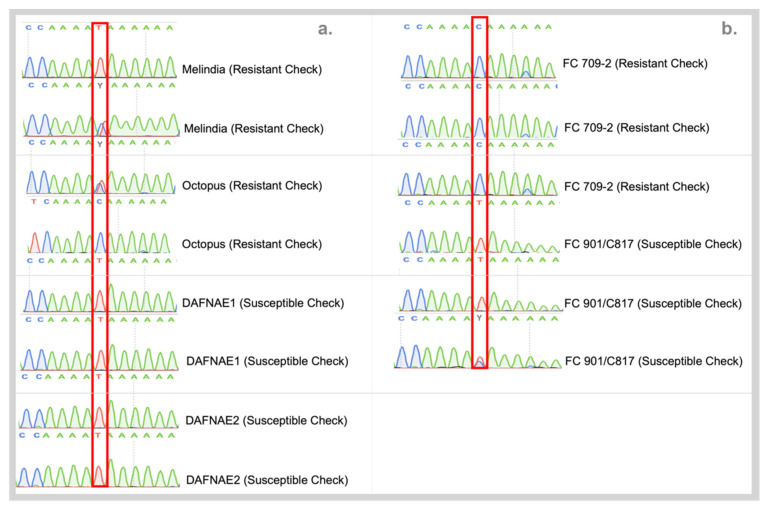
(**a**) Chromatograms from Sanger sequencing of RsBv1 on commercial checks target show predominance of the C allele in resistant plants. (**b**) Validation of RsBv1 on USDA plant material confirming the distinctive allelic status at 9,000,093 bp on Chromosome 6 of sugar beet.

Sanger sequencing of the promising SNP candidates from preliminary validation based on HRM, incorporating all tested checks from USDA with biological replicates resulted in the confirmation of RsBv1 being able to distinguish the two groups. The resistant material was predominantly showed the C allele in the homozygous form whereas the susceptible material had the T allele in the homozygous form at position 9,000,093 bp on chromosome 6 (Figure 5). To further build the association between RsBv1 and *Rhizoctonia* resistant phenotype, a genotyping assay was developed to screen larger germplasm from other sources.

### 3.4. Association between RsBv1 Marker Genotype and Rhizoctonia Resistance in Strube Research Material

**Figure 6:**
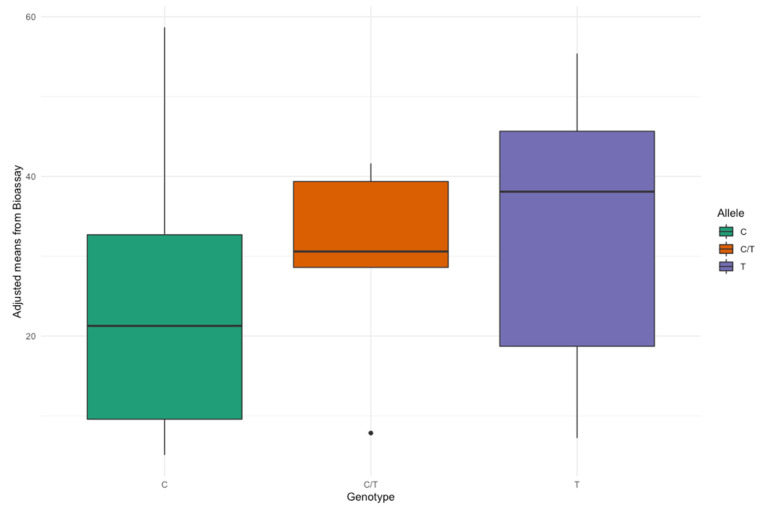
Boxplot between the three marker genotypes of RsBv1 (Chr6, 9,000,093 bp) and adjusted means obtained from *Rhizoctonia* bioassay of 62 lines.

The availability of a range of breeding materials for *Rhizoctonia* resistance allowed us to correlate the marker genotypes obtained for 157 plants from 62 breeding lines with their disease scores obtained from the adjusted means of the percentage of infection (Supplementary Materials Figure S4). Figure 6 shows the differences observed between the groups while comparing the adjusted means from the bioassay with the allelic status—C/C, C/T, and T/T.

Additionally, a generalized linear model which was run to see the association between the adjusted means and the marker genotypes showed that the marker is significantly associated with *Rhizoctonia* (*p* < 0.01). From the adjusted R^2^ of the model, the marker RsBv1 explained 10.99% of the phenotypic variation. As the heterozygous group was small (only six cases), we also did a *t*-test for the adjusted means between C and T (excluding the heterozygous genotypes), which showed a significant difference (*p* < 0.01). Both these results indicated that the marker is significantly associated with *Rhizoctonia*.

We further compared the marker genotypes with the phenotypic bioassay data and the genetic background of the tested lines. Among the 62 tested lines, the marker genotypes of 35 lines fit their bioassay data and genetic background while the marker genotypes of 16 lines did not fit for their bioassay data and/or genetic background. Eleven lines were unknown because they either lacked an SNP call or genetic background. Therefore, the matching correction rate predicted by the marker RsBv1 was calculated as 35/(35 + 16) × 100 = 68.6%.

### 3.5. Further Validation of RsBv1 Based on Genotyping the USDA and SBSI Materials

The association of RsBv1 with *Rhizoctonia* resistance was further confirmed on additional germplasm from USDA and SBSI (Table 2). The bold values represent the count of individuals obtained in each category along with a percentage calculated based on the total number of individuals tested. The resistant material always showed a high frequency of the C allele, and the susceptible material had a high frequency of the T allele. The chi-square test used to analyze the contingency table formed by the evaluated alleles and phenotypes as categorical variables was significant (*p* < 0.05). Thus, *Rhizoctonia* resistance could be linked to the higher frequency of the C allele in the RsBv1 position.

## 4. Discussion

The genetic basis of resistance to *Rhizoctonia solani*, the most severe soil-borne disease of sugar beet is complex due to the interplay between several host-pathogen factors and the environment. It is therefore possible that different genes confer resistance in diverse germplasm due to genetic heterogeneity. The quantitative nature of resistance with additive effects of both major and minor genes has been shown in other crops [40]. Thus, this report is directed towards the identification of SNP markers and thereby provides a systematic determination of key resistance genes in response to *Rhizoctonia* infection.

Our study provides information about markers linked to *Rhizoctonia* resistance using two sets of geographically diverse germplasm from Iran and Germany (206 plants from 32 genotypes) to initiate the identification of SNP markers using RAD-seq. One of the foremost factors determining the success of marker discovery and association study is the plant material and the phenotyping variation. Here, although we used geographically diverse germplasm, a priorly established phenotyping method formed the basis for the study (Buttner et al. 2004). It is worth mentioning that the strain for inoculation (AG-2-2-IIIB), growth cycle, and scoring based on the percentage of infected roots were comparable among the locations (Iran and Germany, Figure 1 and Figure 2) allowing the selection of ideal plant material for DNA sequencing.

Key steps in the release of new commercial resistance varieties include trait mapping and trait introgression [41]. Both these steps are highly reliant on the development and efficient use of molecular markers like SNPs. SNP data is high-dimensional and requires appropriate processing to obtain meaningful associations between phenotype and a DNA marker. PCA-biplots allow visual appraisal of large data matrices. The association analysis was independently carried out for the two datasets and later combined to understand the contribution of mutually occurring SNPs that underlie resistance in different genotypes. Our strategy of filtering candidate SNPs using the likelihood ratio test (Fisher’s test) and the use of different germplasm resulted in a subset of manageable SNP targets. This gave us the chance to study the population structure and reliance of SNP components using the invaluable PCA- biplots. This is to be emphasized because the use of such plots is rare due to data obscurity in the event of numerous SNPs/samples [39] (Figure 3). Other candidates from the PCA-biplot can be correlated with the sequences provided (Supplementary Materials Table S1). The knowledge of these additional loci along with the genome sequence of sugar beet may be used in the marker development for resistance to different *Rhizoctonia* strains as well. Additional markers could be useful to track other novel sources of resistance to *Rhizoctonia* in specific plant germplasm.

Next-generation sequencing methods along with data analyses often result in a large number of associated SNP candidates. However, to have a dialogue with the breeders and initiate the translation process, we proceeded with the use of a streamlined molecular validation strategy to confirm the association of SNPs. The SNP marker, RsBv1 identified in this study can not only be used to guide the screening of sugar beet germplasm for *Rhizoctonia* resistance since its association was confirmed in broad germplasm from the USA, Iran and Germany but also to significantly improve the accuracy and efficiency of its introgression of resistance genes in germplasm management. Moreover, the rhAmp allelic discrimination assay developed and validated here is a PCR-based assay, robust in detecting the resistance allele and separating heterozygotes from homozygotes and thus can be used in the high-throughput selection of *Rhizoctonia* resistance being scalable in 96-, 384- and 1536-well plates.

**Figure 7:**
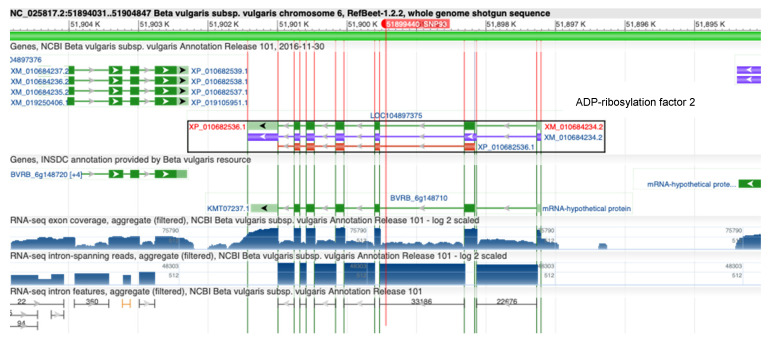
Genomic context of RsBv1 in the RefBeet1.2.2 sugar beet reference genome [29]. It is located within the intron of LOC104897375 coding for ADP-ribosylation factor 2.

Analysis of genomic context of the identified RsBv1 showed that it is located in an intron of the gene coding for ADP-ribosylation factor 2 (Figure 7). ADP-ribosylation factors (ARFs) belonging to the Ras superfamily of small GTP-binding proteins (GTPases) carry out diverse molecular and physiological roles in eukaryotes [42,43]. The ARFs have been mapped and identified in several plant species including *Arabidopsis thaliana*, rice, tomato, potato, maize, carrot, wheat, tobacco, and barley. Recently, ARF proteins have been shown to play roles in conferring tolerance to biotic as well as abiotic stresses in crop plants. In an interesting report, transgenic tobacco plants over-expressing OsARF1 have shown spontaneous expression of pathogenesis-related (PR) genes that reduced susceptibility to fungal pathogens [44]. In yet another study aimed at identifying virulence-associated miRNA-like small RNAs (milRNA) and their targets in response *to R. solani* infection in wheat, ADP-ribosylation factor emerged as one of the targets [45]. While the functional relevance of RsBv1 might be doubted due to its presence within an intron, it is important to note that these classes of genes (ARFs) show variations in their size and intron/exon numbers. They could be ideal candidates for alternative splice forms which are also worth exploring [46].

## 5. Conclusions

One of the fundamental goals of breeding programs is the development of robust markers for marker-assisted selection and the release of resistant cultivars. The RsBv1 marker identified and largely validated in this study allows for rapid screening of multiple genotypes providing alternatives to labor-intensive phenotyping methods. The utility of one such valuable and robust marker was demonstrated through validation on diverse germplasm with both quantitative and qualitative phenotypes and using multiple SNP detection technologies providing an advantage to breeders interested to incorporate molecular selection tools. In addition, the association study resulted in the identification of key SNPs due to the sequencing of geographically diverse germplasm. Each or all of these candidate SNPs reported in this study can be used in the wide screening of *Rhizoctonia* resistance as a panel.

## Figures and Tables

**Table 1:** Description of plant material from SBSI, Iran used for phenotyping and SNP discovery. * indicates the crosses.

ID	Description	No. of Samples	Replicate	Year
S1-930051	Self-pollinated	4	2	2018
S1-89016	Self-pollinated	6	2	2018
S1-92282	Self-pollinated	5	2	2018
S1-92366	Self-pollinated	7	2	2018
S1-92415	Self-pollinated	4	2	2018
S1-92515	Self-pollinated	4	2	2018
SB-39	Self-pollinated	5	2	2018
B-09	Self-pollinated	3	2	2018
SC4*P1 (SC MH41 * SHR01-P.12)	Hybrid	4	2	2018
SC1*P2 (SC MH070 * SHR02-P.4)	Hybrid	5	2	2018
SC2*P2 (SC MH076 * SHR02-P.4)	Hybrid	1	2	2018
SC5*P4 ((7112 * SB36) * S1-88605)	Hybrid	2	2	2018
SC3*P5 (SC MH7 * F-8726)	Hybrid	4	2	2018
SC1*P6 (SC MH070 * F-8738)	Hybrid	4	2	2018
SC4*P7 (SC MH41 * SB27)	Hybrid	2	2	2018
P4 (S1-88605)	Pollinator	5	2	2018
P5 (F-8726)	Pollinator	5	2	2018
P6 (F-8738)	Pollinator	8	2	2018
P7 (SB27)	Pollinator	2	2	2018
SC3 (SC MH7)	Single crosses (MS)	7	2	2018
SC4 (SC MH41)	Single crosses (MS)	5	2	2018

**Table 2:** Contingency table based on rhAmp genotyping of RsBv1 on resistant and susceptible material from USDA and SBSI. The chi-square value provides a measure of the correlation between the categorical variables (the phenotype and the allele of the SNP in each case), and the *p*-value of the statistical test resulted significant (*p* < 0.05).

Source		C	T	Chi-Square	*p*-Value
USDA	Resistant	38	2	51.58	<0.00001
	(*n* = 20)	95%	5%		
	Moderately resistant	32	0		
	(*n* = 16)	100%	0%		
	Susceptible	11	23		
	(*n* = 17)	32.4%	67.6%		
SBSI	Resistant	63	45	18.11	<0.0001
	(*n* = 54)	58.3%	41.7%		
	Susceptible	29	71		
	(*n* = 50)	29.0%	71.0%		

## Data Availability

All raw data related to this study have been deposited in the European Nucleotide Archive (ENA) and linked to the project PRJEB48596.

## Supplementary Materials

The following are available online at https://www.mdpi.com/article/10.3390/biology11010049/s1, Supplementary Figure S1: *Rhizoctonia* scoring scale for phenotyping done in Iran. Supplementary Figure S2: Circular Manhattan plot from association analysis showing significantly associated SNPs from Iran dataset distributed across the EL10 sugar beet reference genome. SNPs highlighted in green have a *p*-value < = 0.05 and SNPs highlighted in red have a *p*-value < = 0.01. Supplementary Figure S3: Circular Manhattan plot from association analysis on Strube Research germplasm highlighting mutually occurring significant SNPs with the association analysis from SBSI dataset. SNPs highlighted in red were identified as strongly discriminating the contrasting phenotypes with a *p*-value < = 0.01. Supplementary Figure S4: Adjusted means of 62 breeding lines (including standards and controls) from *Rhizoctonia* bioassay. Supplementary Table S1: Sequences of top 60 significantly associated SNPs discriminating resistant and susceptible phenotypes from the bioinformatics analyses. Supplementary Table S2: Ruppel scores (Scholten et al. 2001) of USDA releases from field rating done in 2018 used for validation of RsBv1. Supplementary Table S3: rhAmp allelic discrimination assay details for RsBv1 testing.
