# Supplementary material for: Development of an SNP Assay for Marker-Assisted Selection of Soil-Borne Rhizoctonia solani AG-2-2-IIIB Resistance in Sugar Beet
Source: Biology (Basel). 2021 Dec 29;11(1):49. doi: 10.3390/biology11010049 (PMC8772932; doi:10.3390/biology11010049)
Supplement: Supplementary file 1 [file biology-11-00049-s001.zip › biology-1511339-supplementary.pdf]

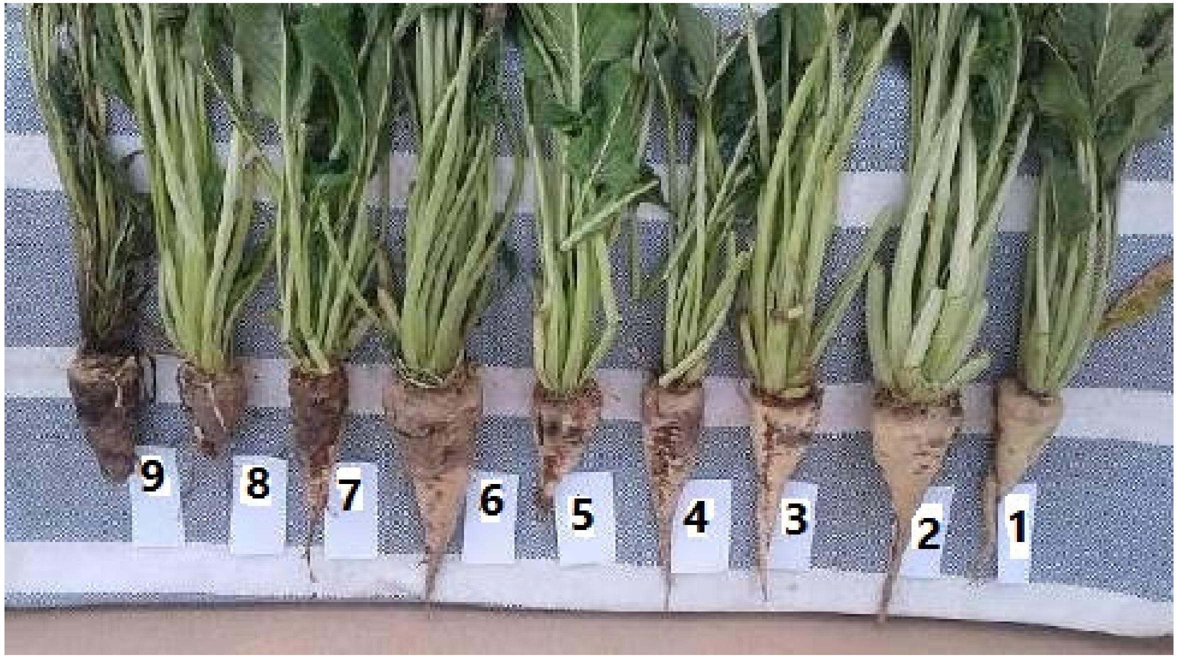

Supplementary Figure S1: *Rhizoctonia* scoring scale for phenotyping done in SBSI, Iran.

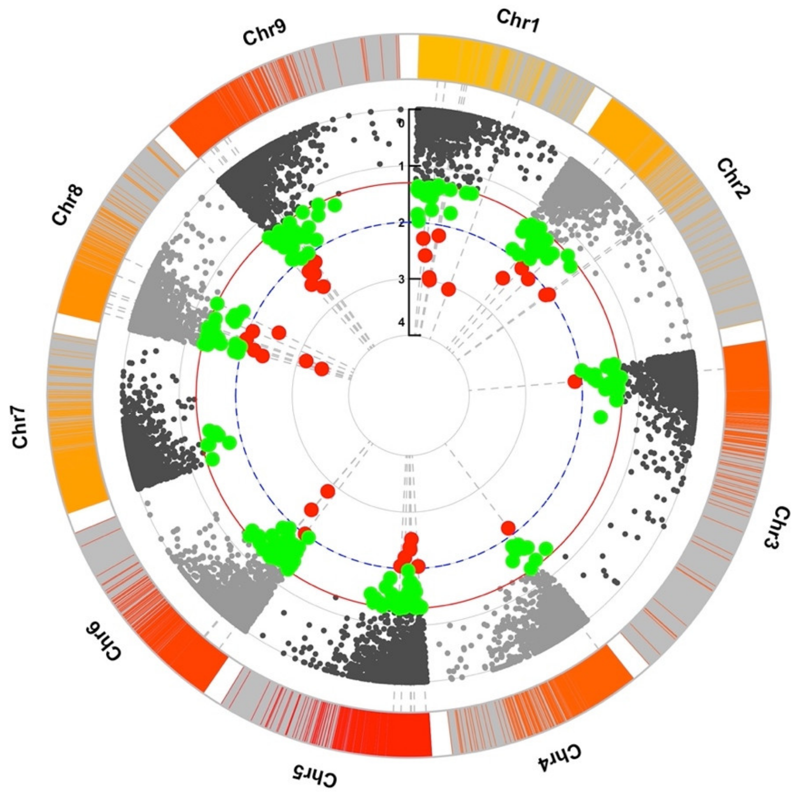

Supplementary Figure S2: Circular Manhattan plot from association analysis showing significantly associated SNPs from SBSI dataset distributed across the EL10 sugar beet reference genome. SNPs highlighted in green have a p-value <= 0.05 and SNPs highlighted in red have a p-value <=0.01.

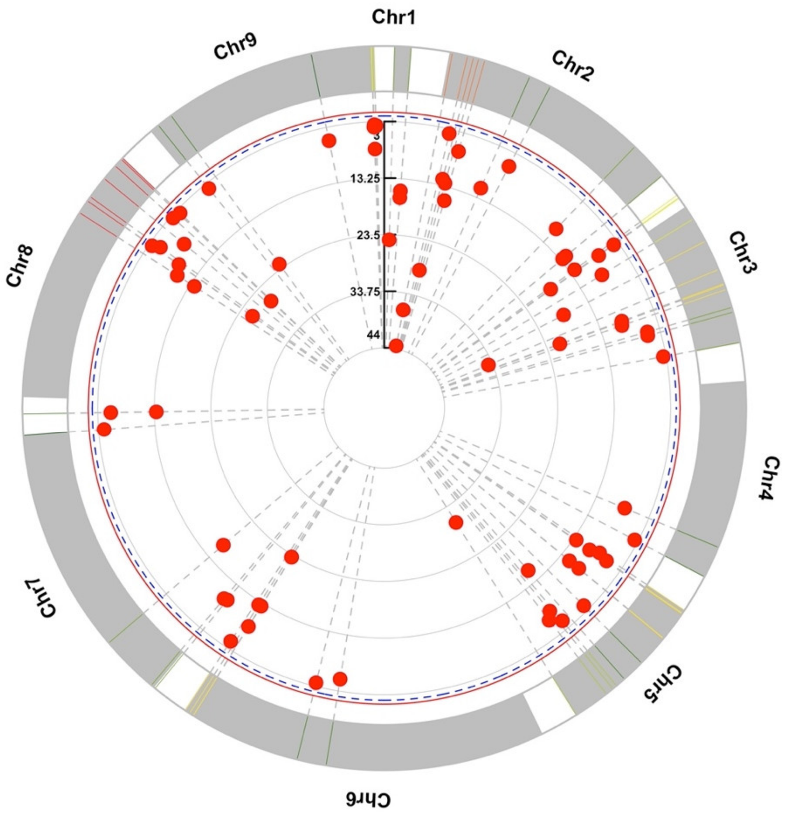

Supplementary Figure S3: Circular Manhattan plot from association analysis on Strube Research germplasm highlighting mutually occurring significant SNPs with the association analysis from SBSI dataset. SNPs highlighted in red were identified as strongly discriminating the contrasting phenotypes with a p-value <=0.01.

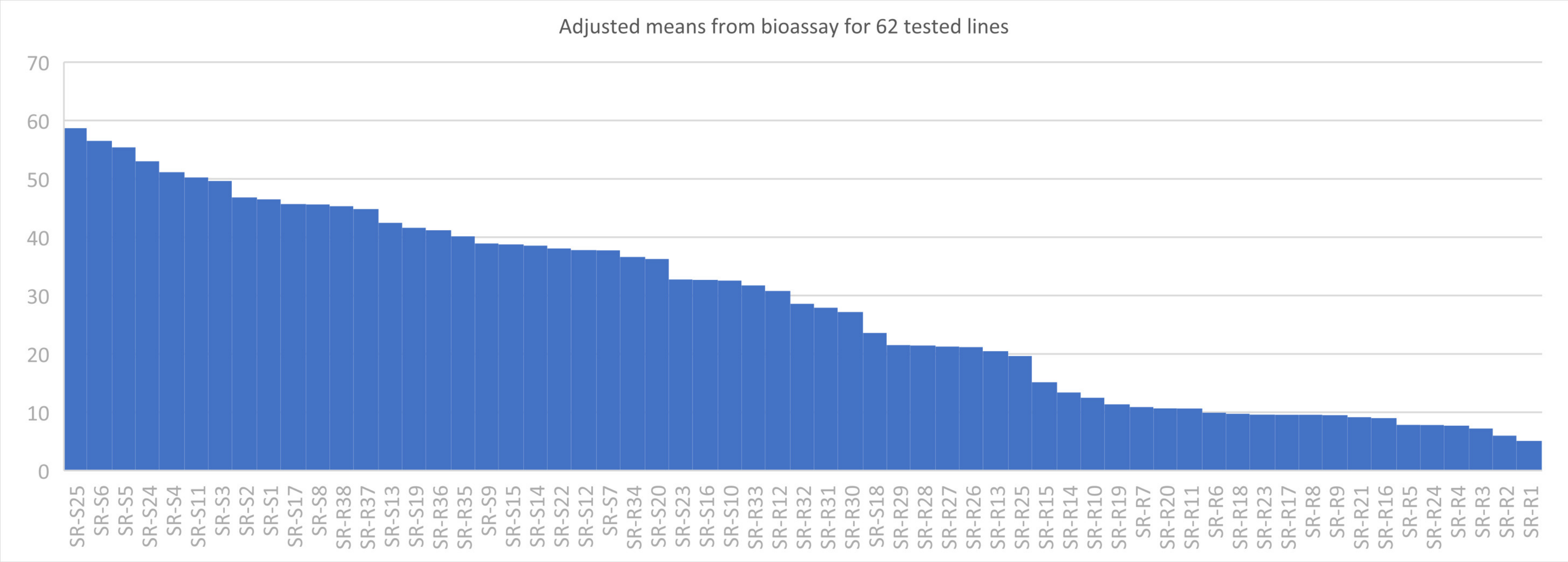

Supplementary Figure S4: Adjusted means of 62 breeding lines (including standards and controls) from *Rhizoctonia* bioassay.

Supplementary Table S1: Sequences of top 60 significantly associated SNPs discriminating resistant and susceptible phenotypes from the bioinformatics analyses.

SNP	Sequence	SNP position	Reference allele	Alternate allele
SNP26	TGCAGTGATCTCTTTCATGTTGTATTGCTCTATAGCGTACACGACCATGTTGGCACTCCAACCACTTGA	61	A	T
SNP32	TGCAGAAGATGGAAGGCAGCAAAATATTTCTTCATACCATCATCGTCATAATCATCTAAATATCTGGGG	66	G	A
SNP125	TGCAGATCATACAAGGTATTCCAAATGCAACTGCTGTGCAGCTTGATGTGTCAGACCAAGAGAGTTTGC	6	A	G
SNP116	TGCAGGTTCGTCTACCACTAAACGTCTTCTTACCAGTTCTGTTACAGATCGGAAGAGCGGTTCAGCAGG	9	C	T
SNP4	TGCAGAGCCGCTACTCACCGCTGCCTGTTCTTATCTCTCCTCTGTTGAAGTTCCTTTTTCTTCTTTTAT	62	T	C
SNP81	TGCAGTATGTACACAAAAGGATCAGCCAATCTTAGACTACAGCTAACCAAGCAAAACAAAGAACTTCAA	30	T	C

SNP126	TGCAGTGGTACCGAGTGTGCCCTTTGGTTTTTGCAATACTGCATAATTTTACCTGGTTTGCCTCCATTA	12	C	T
SNP47	TGCAGAAGAACATATATCATTAGCTTACCAAAATTACAGATCGGAAGAGCGGTTCAGCAGGAATGCCGA	20	T	C
SNP62	TGCAGAAGCACAAGATGAAGAGGAGCAAGATGAGGAGATGCTGCGCAGTTCGTGCTGCAACCAGAGTAC	45	G	A
SNP75	TGCAGTTTTGGGACACTTGACTTGTATATCAACTGAACTTTCGCCACAAGTTTGGTACAGATTACTCAG	13	A	G
SNP43	TGCAGTTTTGGGGCACTTGACTTGTATATCAACTGAACTTTGGCCACAAGTTTGGTACAGATTACTCAG	21	C	A
SNP53	TGCAGTCAGAAGAAATGCAGCAGCCCAGGTGATCGCCAGACCCAAGGGAACCTGACAACAGCTTATTAG	34	C	T
SNP16	TGCAGATTTTTCTCCACGCATCTAGCAAAACTCTGAAAGCTACCAGTGGTGAATTGCTGATTTTTCAAC	27	A	T
SNP19	TGCAGTCTTGTGGAGATAAGGCTGGATTAGCGTGTTTTGAATATTTACAGATCGGAAGAGCGGTTCAGC	30	G	C
SNP86	TGCAGCTGAAGCACGACCCACAGACCACCAAGGAGCATGGGCCTTGTGACATATACAGCCACTAGCATC	51	A	G

SNP21	TGCAGCCAATTCACTGGTGGGCTTTTGCTGATACAATGTTTCATAGAAAATCCTCAAAGGATCTCTCTG	52	C	T
SNP91	TGCAGTTTTGTTAGATTCTGAAGATTCATGATGCCAATAAATTGACATTTATTGTTCTTGATTGAATTG	66	A	C
SNP18	TGCAGTTATGACATGGCAACATGCATAGCAGAAAAATTACAGATCGGAAGAGCGGTTCAGCAGGAATGC	31	G	C
SNP15	TGCAGTAAAAAAGTTCCAAAGATGGGAATGGCACCTTCTTTTTTACAGATCGGAAGAGCGGTTCAGCAG	32	C	T
SNP71	TGCAGACTATAATGCAAATCTTAGTTGCTTCCCTCTTTCTCTTCTATTATTCTATGAGGTGTGATGATA	51	T	A
SNP17	TGCAGTCTAAGTACTTGATATTTATTTGCTTCCTAAAATACACCTTTCACTGTAAATTATGTGAGTATT	27	T	C
SNP113	TGCAGGCCTTGGCAATCCTGAGCTTTCTCATTACAAGTTGAACCTACAAGAAAGTGCTTCTAACAGAAG	47	C	A
SNP72	TGCAGGCATAGAGAGGAATGATGCAAATTGTCTGAGAAGGCTATATTACAGATCGGAAGAGCGGTTCAG	29	T	C
SNP58	TGCAGATCCAAAGACAAAAGAGGATGTACACAAAGTGCCTAACAGGGAGACGCTCACCTTGTAAGTTCT	51	C	A

SNP20	TGCAGTGATCGCTCCCAAGAAGGCCTGGACAGGGGATGGGGTTTTAGATTCAATCGAGACTGATCCAAT	10	C	T
SNP34	TGCAGCAGGAGTTGTCTGCTGCTAAAACAGAAGTAAACAGAGTTGAGTCAAGTATGGCTGATGCATTAT	14	G	A
SNP29	TGCAGCCGCGACTAGCTGCACCGGTGGAGTACCAACAGAGATTTGCATAAGGAAATCTCCCTCATTAGG	63	C	G
SNP1	TGCAGCCGTTTTCTTACTTTTCCTACTCTACTATCTTTCCAGCCACAACGAGGCAAAAACACCAAACTC	24	T	C
SNP55	TGCAGTTACATTTTTGCCCTCTTTATTCGTCGCTTTCTCCACTATTTCACGTTTCATTCCAAGTATCCA	57	T	A
SNP12	TGCAGATGATTTGGCTTGCTCCCTGAACCCTTCCTAGAAGGTGGCAGGAAGTTACAGATCGGAAGAGCG	6	A	G
SNP23	TGCAGTCTGATGACCATTGTTGCAACTCTTGTGAAGAAGTTCGTGAGGCTTACAGAAAGAAGGGCTGGG	65	C	A
SNP50	TGCAGAAAAACTTGACTTCAGAAGATTTACTACCCTCTTTGTGGGATAAATGCAAGGTGAGCAAAATCA	26	T	C
SNP96	TGCAGATTATGAAATACACATGTATGATTGTATAAATTGATGGTATATTGTGGTGTATTTTTAGGTGGA	17	C	T

SNP24	TGCAGAAAATGTATTTCCTTCCTTCCAACATTTTCCCATATCAACTTCATTACAGATCGGAAGAGCGGT	30	A	G
SNP42	TGCAGAAATTGCTTTACGGTTATCCAGATAAACAAGAAGATGACTAAAATATTCTCTAACCATGAGGTC	54	C	T
SNP69	TGCAGCATCAGCGATGCTTATTTGCATTGAGTGGATTACAGATCGGAAGAGCGGTTCAGCAGGAATGCC	11	G	A
SNP110	TGCAGCATCAGTGATGCTTATTTGCATTGAGTGGATTACAGATCGGAAGAGCGGTTCAGCAGGAATGCC	12	T	C
SNP64	TGCAGCATCTGACATTTGAGAAACTGGTGAAATATTTCAGCACATATTCTTGCGACAACAATGCGCTCA	65	G	A
SNP8	TGCAGACTACATAAATGCACGGGGGTTAGAATATATGTGTGCCTCTGAAAGACTGAAACAACATTTTAC	69	C	G
SNP67	TGCAGATATCTCAAAATCTGGATAACAGAAAATCAAAGATCCGTGGATGCGTATTACAGATCGGAAGAG	12	C	G
SNP59	TGCAGATATCTGAAAATCTGGATAACAGAAAATCAAACATCCGTGGATGCGTATTACAGATCGGAAGAG	38	C	G
SNP56	TGCAGCCACCTATTCCATTTTCATGGACGTATCACTCTGACCTCATTAGAGCTGCTCATCAGACTGGAG	29	G	T

SNP130	TGCAGAAAGCTTCAGCTTTGCTCTACCTAAATTTCAATCTTTCTTGAGTTCGTAGGGGTTCAAGCCTGC	23	C	T
SNP104	TGCAGATATGGAATCGAGAGTGAATGAAAATGTATGGCAACGATTTGGGATTGAGCAACATCCTCCGCG	41	C	A
SNP49	TGCAGGGTTGCTTGGAGCTTGTTCGCCAGGTACTGGTGCTCGGATTATTGCTTTGGTGGGTGGTCCTTG	67	T	A
SNP41	TGCAGAGTTGTCTTTCCTGCAACTCTAATCTTTAGGGAAATCCCCAATGATAGGCTCCTATTTTTTAGA	24	T	C
SNP60	TGCAGCAAGGACAACTCCGTGCACATTGGATGTTGGGCAAAAACCAATAGCTGACATTTGATTCAGCAA	18	C	T
SNP46	TGCAGAAGTTTGGATTCTGAGAATAATAGTTGCTATGTTTTTATGGTGGAAATTCTCAATACTCCGAA	49	G	A
SNP51	TGCAGGATATTGCATTTCATCGGTATAATGGGGTTCACGTTATTGGAAGATGCACAGGAGAATTTGTAC	55	C	T
SNP57	TGCAGCTATGGTGCATAAACAAAAGGGGTCCAGTTTTTGCTGCAATGTTTACAGATCGGAAGAGCGGTT	41	T	C
SNP70	TGCAGCCCAGATTCCCTCCTTAGAACAACACTCATTTATTAGGTTACAGATCGGAAGAGCGGTTCAGCA	14	C	T

SNP119	TGCAGAACTTCGTTTACTCTTATGATTCCCCGAGCTAGCTTTGCAACATCCATGGAAAGGGAGTTGGAA	56	A	C
SNP109	TGCAGGTACTTACGGGCGAAGCAAGTCTACCATCAGTCCCTTTTGAGAAGCCTGCCTTTGTATGGCCAG	14	G	A
SNP94	TGCAGGAGTCGATTCCAACCAACGCCACAAATTTTAGATGGTTGCATTTCACTGTTGGCAGAAGGTATT	10	C	T
SNP22	TGCAGTATAGAATGATAATTCCTAGTCTTGGTGATAATGTCCGGGTAACTAAAATCTAATTCCTAGTTT	43	G	A
SNP7	TGCAGAAAAAAGCGCAGATGAAAGAAAAGAAACATATTCTTTACAGATCGGAAGAGCGGTTCAGCAGGA	13	C	T
SNP27	TGCAGCAAGTTCAATACCAACCGTTACTATATCCAAGATTTCTAAAAGGGGATAGAAGTAGCTTTACAG	41	T	C
SNP76	TGCAGGTACATATGATAATTGATATGGCAGAAATGATTGCTTCAAGTAGTGTCCATTTAGGGAATTTTA	38	T	A
SNP93	TGCAGATACAAGATATACCAAAACAAAAAACAAAAAACATAGCATATCTTGTAAAAGATGGATCTAATT	24	C	T
SNP102	TGCAGCACAAATAAATGGCGTGCTTTATGTTGTTGGTGGATATGATGGAAGTGTATACCTAGGGTAAGC	23	A	C

Supplementary Table S2: Ruppel scores (Scholten et al., 2001) of USDA releases from field rating done in 2018 used for validation of SNP93.

Release	*FC709-2*	*FC709-2*	*FC709-2*	*FC703*	*FC703*	*FC703*	*FC901/C817*	*FC901/C817*	*FC901/C817*
genotype	20121034	20121034	20121034	20041005	20041005	20041005	20141002	20141002	20141002
Replicate	Rep1	Rep2	Rep3	Rep1	Rep2	Rep3	Rep1	Rep2	Rep3
field plot	2002	2040	2070	2001	2041	2063	2000	2034	2064
Plant 1	1	3	1	1	3	4	5	6	5
Plant 2	1	2	2	2	3	3	6	5	1
Plant 3	1	2	1	1	2	3	5	7	2
Plant 4	1	1	2	1	2	3	5	6	4
Plant 5	2	2	1	3	1	3	5	5	4
Plant 6	1	1	3	4	3	3	4	7	3
Plant 7	2	2	2	1	2	4	6	4	4
Plant 8	0	4	2	3	4	4	6	6	4

Supplementary Table S3: rhAmp allelic discrimination assay details for SNP93 testing.

**Primer**	**Sequence**
Allele Specific Primer 1 :	TGCTGCAGATACAAGATATATCAAAAC
Allele Specific Primer 2 :	ATGCTGCAGATACAAGATATATCAAAAT
Locus Specific Primer :	GCAAGTCTGAATTTCTGATCTCATCTTTA
